# Point prevalence survey of antibiotic use in Mexican secondary care hospitals

**DOI:** 10.1371/journal.pone.0315925

**Published:** 2025-01-03

**Authors:** Federico A. Zumaya-Estrada, Celia M. Alpuche-Aranda, Hilda Ivonne Huerta Icelo, Felipe D. Neri-Estrada, Verónica M. Calixto Silva, Haydee E. Quiroz Escoriza, Jesus Ulises Garza-Ramos, Pedro J. Saturno-Hernandez

**Affiliations:** 1 National Institute of Public Health of Mexico, Center for Infectious Diseases Research, Cuernavaca, Morelos, Mexico; 2 Pharmacovigilance Unit, General Hospital of Cuernavaca "Dr. José G. Parres", Health Services of Morelos, Cuernavaca, Morelos, Mexico; 3 Department of Teaching, Research and Training, Women’s Specialty Hospital, Health Services of Morelos, Yautepec de Zaragoza, Morelos, Mexico; 4 National Institute of Public Health of Mexico, Center for Evaluation and Surveys Research, Cuernavaca, Morelos, Mexico; North Carolina State University, UNITED STATES OF AMERICA

## Abstract

**Introduction:**

Tackling the inertia of growing threat of antimicrobial resistance (AMR) requires changes in how antibiotics are prescribed and utilized. The monitoring of antimicrobial prescribing in hospitals is a critical component in optimizing antibiotic use. Point prevalence surveys (PPSs) enable the surveillance of antibiotic prescribing at the patient level in small hospitals that lack the resources to establish antimicrobial stewardship programs (ASP). In this study, we analyzed antibiotic use at two public secondary care hospitals in Mexico using PPSs.

**Methods:**

Following WHO methodology, we conducted four cross-sectional PPSs on antibiotic use in two public secondary care facilities in Mexico: two surveys in a women’s specialty hospital (H1) and two in a general referral hospital (H2). We collected data from clinical records of all patients with active antibiotic prescriptions (APs) across the medical, surgical, and mixed (MIX) wards, and intensive care units (ICUs). Descriptive statistics were computed to analyze the PPSs data using Stata.

**Results:**

The PPSs collected data on 127 patients, and 283 active APs. The prevalence of antibiotic use was 60.4% (H1, *n* = 29/48) and 70.5% (H2, *n* = 98/139). Antibiotics were more frequently used among patients in the MIX wards (H1: 87.5%, *n* = 14/16) and ICUs (H2: 90%, *n* = 9/10). The most frequent patient indications for antibiotic use were medical prophylaxis (H1: 51.7%, *n* = 15/29), community-acquired infections (H2: 42.9%, *n* = 42/98), and preoperative prophylaxis (H1: 27.6%, *n* = 8/29; H2: 23.5%, *n* = 23/98). The APs were mostly empirical (H1: 97%, *n* = 64/66; H2: 98.2%, *n* = 213/217), and parenterally administered (H1: 90.9%, *n* = 60/66; H2: 96.8%, *n* = 210/217). Most clinical records lacked documented post-prescription reviews (H1: 82.8%, *n* = 24/29; H2: 98%, *n* = 96/98). Preoperative prophylaxis was predominantly administered as multiple doses for more than one day. Penicillins with extended-spectrum (24.2%, *n* = 16/66), aminoglycosides (22.7%, *n* = 15/66), and first-generation cephalosporins (16.7%, *n* = 11/66) were the most prescribed antibiotic classes in H1, while third-generation cephalosporins (35%, *n* = 76/217), fluoroquinolones (14.3%, *n* = 31/217), and carbapenems (13.4%, *n* = 29/217) were the most prescribed in H2. No hospital had formally established ASP.

**Conclusions:**

This study shows high prevalence rates of antibiotic use and variations in commonly prescribed antibiotic classes in public Mexican secondary care hospitals, along with shared practices in broad-spectrum antibiotic prescription. PPS-based surveillance enables the identification of specific targets to optimize antibiotic use according to the healthcare needs of patients in each hospital and facilitates comparative evaluations across hospitals.

## Introduction

Antibiotics are the cornerstone for both preventing and treating bacterial infections [1]. Nevertheless, the misuse and overuse of antibiotics across environmental, agricultural, and healthcare sectors are accelerating the emergence of antibiotic-resistant bacteria. The rising phenomenon of antimicrobial resistance (AMR) is rendering available antibiotic options ineffective, making it increasingly difficult to prevent and treat infections [2,3]. Addressing the inertia of the AMR threat requires a fundamental shift in antibiotic utilization, ensuring judiciously use wherever necessary [4]. Antibiotic use is a routine and essential component of daily hospital practice, but up to half of the patient antibiotic therapies may not meet recommended clinical practice guidelines [5]. The antibiotics misuse not only leads to healthcare cost overruns but also elevates the risks for patients, including serious adverse drug reactions or developing multidrug-resistant (MDR) infections associated with higher mortality rates compared to infections caused by susceptible bacteria [6–8]. Consequently, the surveillance of antibiotic use in patients is crucial for developing hospital antibiotic stewardship interventions aimed at improving the opportunity to provide effective and safe antibiotic therapies [9]. In 2018, the Mexican government published a National Action Strategy against Antimicrobial Resistance, which includes implementing routine surveillance antibiotic consumption and use in public secondary and tertiary care hospitals, following standardized methods [10]. However, consistent data on antibiotic use in the Mexican hospitals remains lacking [11]. Such gaps in monitoring antibiotic use are common in hospitals across low- and middle-income countries (LMICs) [12], due to resource constraints and the high workloads faced by healthcare worker [13,14]. A viable alternative for hospitals encountering challenges in antibiotic use surveillance is to collect patient prescription data at a specific point in time, using point prevalence surveys (PPSs) [15,16]. The PPSs have provided detailed insights into antibiotic prescribing on hospitalized patients across healthcare settings worldwide, including large hospitals globally [17], from Europe [18], Latin America, Caribbean [19–21], and in Mexico [22]. Most of PPS studies conducted in Mexican hospitals have primarily focused on large academic and tertiary care hospitals [20–22]. However, a significant portion of healthcare is delivered in small community institutions. As of 2024, 96% of operational hospitalization units in Mexico are secondary care facilities [23]. These hospitals provide a wide range of services, from routine cesarean sections to the management of life-threatening conditions such as sepsis [24,25], which can result in substantial variation in antibiotic use patterns [26]. In this study, we present the results of an analysis of antibiotic use in two public secondary care hospitals in Mexico, conducted using PPSs.

## Methods

### Study protocol

This study is based on cross-sectional PPSs, conducted following the WHO methodology for point prevalence survey on antibiotic use in hospitals (WPPS) [16]. Data on antibiotics for systemic use according to the Anatomical Therapeutic Chemical (ATC) classification system (ATC code: J01) were collected [27]. Topical and antituberculosis antibiotics were excluded.

### Hospital selection

The study was conducted in two public secondary care hospitals in Mexico, anonymized as H1 and H2. These hospitals were selected due to their regional significance in providing public medical and surgical care, as well as their expressed interest in adopting the PPS methodology for antibiotic use surveillance.

H1 is a women’s specialty hospital with a capacity of 50 census beds, while H2 serves as a general referral hospital with 144 census beds. Both facilities are situated in urban areas, one in a municipality and the other in the capital city of a state in Mexico’s Central-South region. Within this state, H1 is the only public hospital specializing in women’s healthcare, whereas H2 is the largest public general referral hospital. As of 2022, H1 and H2 reported approximately 3,500 and 5,900 hospital discharges, respectively [28,29].

### Ethics statement

The Research and Ethics Committee of the National Institute of Public Health of Mexico approved the protocol for this study (CI: 1533). The Subdirectorate of Teaching, Research, and Training of the Health Services of Morelos also approved the protocol and implementation of this study at both participating hospitals (Official Letter No.: DAM/SEIC/DIC/3336/2022). Both protocol approvals for this study included a waiver of informed consent for the collection of data from clinical records, as the patients were not research subjects. This was a non-interventional study; therefore, no experimental changes were made to patient care or treatment regimens. Our methodology did not involve any interaction or direct procedures with human subjects, and no personal data or information that could enable subsequent identification of the patients was collected. All data obtained from clinical records were kept anonymous and confidential.

The procedures for accessing patients’ clinical records and collecting data for the purposes of this study adhered to the provisions of the Mexican Official Standard NOM-004-SSA3-2012, On the Clinical Record, and were directly supervised by the personnel responsible for each hospital.

The authors assume the responsibility to protect individuals’ privacy and maintain the confidentiality of all collected data in accordance with the Declaration of Helsinki and the Regulations of the General Health Law regarding Health Research in Mexico.

### Data collection forms

We used three electronic data collection forms that included the core variables of the WPPS [16]. These forms were designed and managed using REDCap (Research Electronic Data Capture), a web-based platform that enables direct data entry and storage on electronic tablets and facilitates the direct export of captured data to statistical software packages [30]. To minimize the risk of entering erroneous data, we incorporated automatic validations, entry field restrictions, and dropdown menus in the forms. The electronic data collection forms used in this study had also been previously implemented in other Mexican hospitals [22].

### Survey content

Two of the data collection forms included variables to collect information on the hospital’s general characteristics and infrastructure, as well as on its wards providing medical (MED), surgical (SUR), mixed medical-surgical (MIX) services, and intensive care units (ICUs). The third data collection form contained variables to collect anonymous data from clinical records of all patients with active antibiotic prescriptions (APs), including admission date, age, gender, clinical diagnoses, infection types (hospital-associated infections [HAIs] or community-acquired infections [CAIs]), and prophylactic indications (medical prophylaxis [MPs] or preoperative prophylaxis [PrPs]). The third data collection form was also used to collect data on the characteristics of APs, such as the generic names of agents, dosages, dosing intervals, route of administration (parenteral, oral, or other), documented reviews at 48 hours, treatment durations, and the basis of prescription (empirical or targeted by microbiological findings). Additionally, we collected information on microbial culture performance and antimicrobial susceptibility testing (AST) results.

### Survey of capabilities to optimize antibiotic use

We also conducted an online survey through REDCap to investigate the infrastructure, policies, practices, and monitoring and feedback activities for prescribers, using 17 basic indicators recommended by a multinational group of antimicrobial stewardship experts [31]. The implementation of this online survey was included in both protocol approvals for this study and was sent directly by one of the study’s principal investigators to the heads of the Department of Teaching, Research, and Training at H1 and the Pharmacovigilance Unit at H2, who were responsible for completing it.

### Team of observers and training

A multidisciplinary team composed of three observers, supported by medical, nursing, pharmacovigilance, and research staff from the hospitals, carried out the collection of PPS data.

At least one of the principal investigators, who assumed the role of survey coordinator, accompanied the team of observers. The observers attended face-to-face training sessions on PPS methodology, practical handling of the PPS data collection forms, and the procedures for clinical record review and data collection.

### Pilot studies

Pilot studies were conducted in both hospitals following recommendations for rigorous application, based on lessons learned from other Mexican hospitals [32]. The same team of observers conducted pilot studies in both hospitals at least one week prior to the PPS.

### Survey dates

Two PPSs were conducted at each hospital: the first during winter months (January and February 2023), and the second during spring months (April and May 2023).

### Patient selection

On the first day of the surveys, we used the daily hospital census to identify all hospitalized patients up to 8:00 a.m. After reviewing their medical indication sheets, we differentiated patients with active APs (selected patients) from those not receiving antibiotics. The surveys only collected data from the clinical records of the selected patients. The number of selected patients and those not receiving antibiotics were included in the total number of admitted patients in the surveys (admitted patients).

Only the clinical records of patients hospitalized after 8:00 a.m., those who had undergone surgery on the day of patient selection, those receiving outpatient care, and those in emergency rooms were excluded from the surveys.

### Data collection and analysis

Data from the clinical records of selected patients were directly captured on electronic tablets by the team of observers. The collected data were exported directly from REDCap to the statistical software package Stata v17 [33]. The surveys were initiated on Monday and continued for four consecutive days. The observers signed letters of commitment to confidentiality prior to assuming their roles as clinical record reviewers. All patient data collected during the PPSs were anonymized. No personal or identifiable patient information was extracted from the clinical records. The coordinator supervised the clinical record review and data entry procedures, ensuring adherence to the protocol. The coordinator always remained available to answer observers’ questions in real time.

The data from both PPSs conducted at each hospital were consolidated. Descriptive statistics (absolute and relative frequencies, medians, and interquartile ranges [IQR], as appropriate) were calculated for discrete quantitative variables and for categorized continuous quantitative variables using Stata v17 [33]. The prevalence of antibiotic use by hospital and wards/units was expressed as percentages, calculated by dividing the number of selected patients by the number of admitted patients.

The classification of antibiotics into the groups: Access, Watch, and Reserve was based on the WHO classification database of antibiotics for the evaluation and monitoring of use (WHO AWaRe) [34].

## Results

### Capabilities to optimize antibiotic use

Neither hospital has established a formal Antimicrobial Stewardship Program (ASP). Furthermore, both hospitals completely lack any type of infrastructure, policies, practices, or activities dedicated to the surveillance and optimization of antibiotic use.

To support prescribers’ decision-making, both hospitals reported having laboratories equipped to perform clinical and imaging studies, as well as clinical microbiology techniques, including microbiological cultures and automated AST.

### Characteristics of selected patients

Our PPSs collected data from 127 selected patients (H1: *n* = 29; H2: *n* = 98) out of a total 187 admitted patients (H1: *n* = 48; H2: *n* = 139). The selected patients occupied beds in wards of general medicine, internal medicine, gynecology, obstetrics, neonatology, general surgery, traumatology/orthopedics, and ICUs for adult, pediatric, and neonatal patients. In both hospitals, most patients were adults (H1: 51.7%, *n* = 15/29; H2: 97.9%, *n* = 96/98). The median age of patients was 24.0 years (IQR: 19.8–24.5 years) in H1 and 49.0 years (IQR: 34.0–64.8 years) in H2. In H1, 82.8% (*n* = 24/29) of patients were female, as it is a hospital dedicated to women’s care. In contrast, most patients in H2 were male (60.2%, *n* = 59/98). The median duration of hospital stays for patients in H1 was 4.0 days (IQR: 1.0–6.0 days), compared to 9.0 days (IQR: 5.0–13.0 days) in H2 (Table 1).

**Table 1:** Characteristics of patients selected for the surveys.

	Hospital	
	H1	H2
Number of patients selected	29	98
Median age (years)	24.0* (IQR: 19.8–24.5)	49.0 (IQR: 34.0–64.8)
Median length of hospital stays (days)	4.0 (IQR: 1.0–6.0)	9.0 (IQR: 5.0–13.0)
**Age groups**	% (*n*)	% (*n*)
Adults (≥18 years old)	51.7 (15)	97.9 (96)
Children (29 days to 17 years old)	17.2 (5)	2.1 (2)
Neonates (zero to 28 days old)	31.1 (9)	-
**Gender**	% (*n*)	% (*n*)
Male	17.2 (5)^†^	60.2 (59)
Female	82.8 (24)	39.8 (39)

Notes:

*Includes all patients aged one year or older.

^†^ Neonatal patients.

**Abbreviations**: H1: Women’s specialty hospital, H2: General referral hospital.

In H1, the most common clinical diagnoses and/or prophylactic indications were obstetric prophylaxis (60.0%, *n* = 21/35), and clinical sepsis (17.1%, *n* = 6/35). In H2, these include pneumonia or lower respiratory tract infections (16.8%, *n* = 20/119), intra-abdominal sepsis (9.2%, *n* = 11/119), and lower urinary tract infections (9.2%, *n* = 11/119) (S1 Table).

### Prevalence and characteristics of antibiotic use

The prevalence of antibiotic use at the hospital level was 60.4% (*n* = 29/48) in H1, and 70.5% (*n* = 98/139) in H2. Across the wards/units, the highest prevalence of antibiotic use was observed in the MIX wards (H1: 87.5%, *n* = 14/16), followed by the ICUs (H2: 90.0%, *n* = 9/10), excluding the MED wards in H1, where only one patient had been prescribed antibiotics (Table 2).

**Table 2:** Prevalence of antibiotic use in the hospitals and their respective wards/units.

Hospital	Hospital			Wards/Units											
				MED			SUR*			MIX			ICUs		
	Antibiotic use (%)	Selected (*n*)	Admitted (*N*)	Antibiotic use (%)	Selected (*n*)	Admitted (*N*)	Antibiotic use (%)	Selected (*n*)	Admitted (*N*)	Antibiotic use (%)	Selected (*n*)	Admitted (*N*)	Antibiotic use (%)	Selected (*n*)	Admitted (*N*)
**H1**	60.4	29	48	100	1	1	-	-	-	87.5	14	16	45.2	14	31
**H2**	70.5	98	139	70.5	43	61	66.1	41	62	83.3	5	6	90.0	9	10

Notes:

*Includes all patients aged one year or older. Antibiotic use: Percentage of patients who received at least one antibiotic out of the total number of hospitalized patients. Selected: Number of patients with active APs. Admitted: Total number of hospitalized patients.

**Abbreviations**: H1: Women’s specialty hospital, H2: General referral hospital. MED: Medical service wards, SUR: Surgical service wards, MIX: Mixed service wards, ICUs: Intensive care units.

Most patients received two different antibiotics (H1: 58.6%, *n* = 17/29; H2: 39.8%, *n* = 39/98). In H2, one out of three patients had been prescribed three or more antibiotics (S2 Table). The APs were predominantly empirical (H1: 97.0%, *n* = 64/66; H2: 98.2%, *n* = 213/217), administered parenterally (H1: 90.9%, *n* = 60/66; H2: 96.8%, *n* = 210/217), and the clinical records lacked documented post-prescription reviews (H1: 82.8%, *n* = 24/29; H2: 98.0%, *n* = 96/98) (S2 Table).

### Most used antibiotics

**Fig 1:**
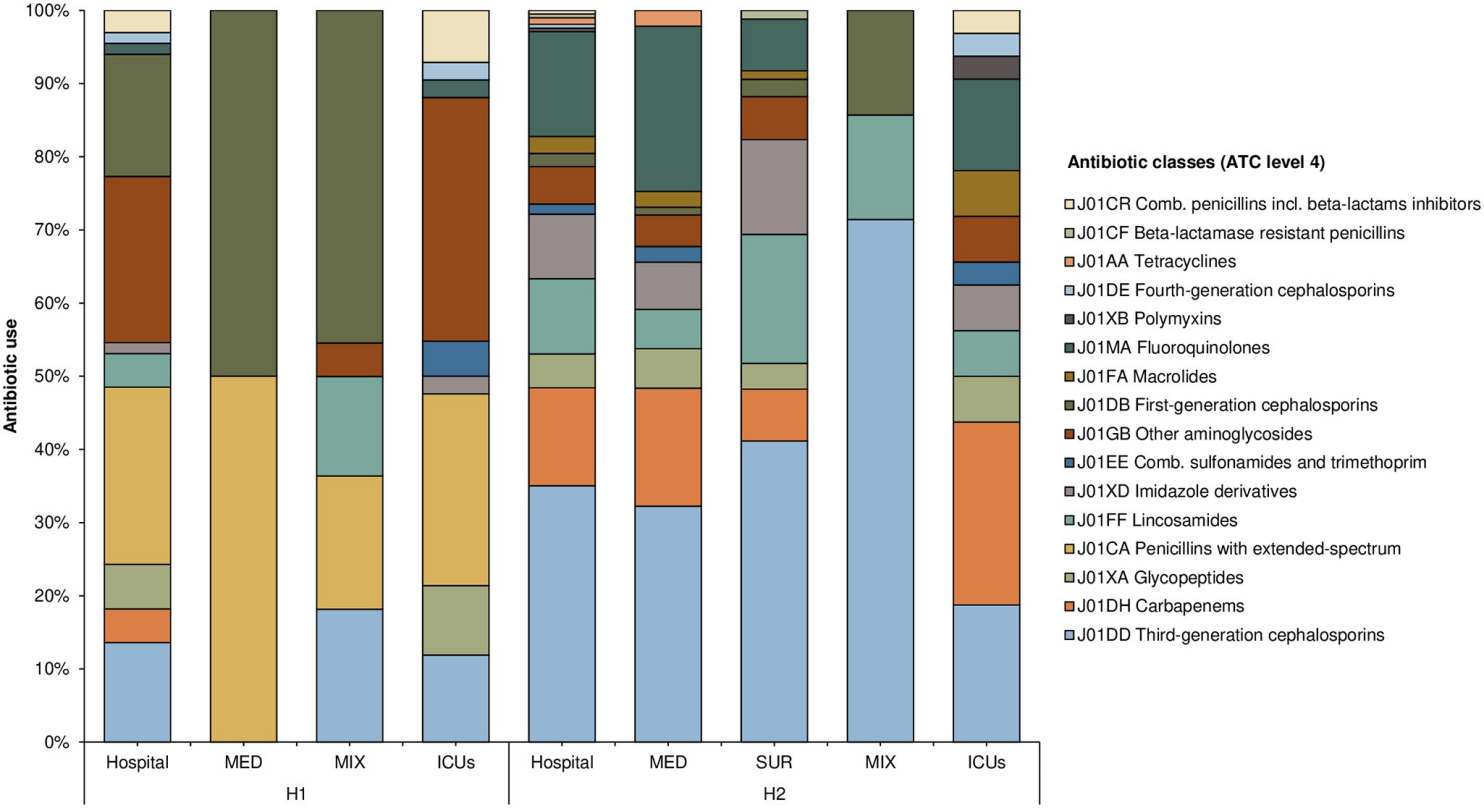
Proportion of antibiotic use (at ATC level 4) in the hospitals and their respective wards/units.

We identified 66 active APs comprising 14 antibiotics (ATC 5) and 11 antibiotic classes (ATC 4) in H1, and 217 active APs involving 24 antibiotics (ATC 5) and 15 antibiotic classes (ATC 4) (Fig 1 and Table 3). The most frequently prescribed antibiotic classes (ATC 4) in H1 were penicillins with extended-spectrum (24.2%, *n* = 16/66), aminoglycosides (22.7%, *n* = 15/66), and first-generation cephalosporins (16.7%, *n* = 11/66), while in H2 they were third-generation cephalosporins (3GC) (35.0%, *n* = 76/217), fluoroquinolones (14.3%, *n* = 31/217), and carbapenems (13.4%, *n* = 29/217) (Fig 1 and S3 Table).

**Table 3:** Antibiotics prescribed in the hospitals and their respective wards/units.

Antibiotics prescribed			Hospital		Global	H1 wards/units*			H2 wards/units			
			H1	H2		MED	MIX	ICUs	MED	SUR	MIX	ICUs
ATC 5 code	WHO AWaRe group	Antibiotic	% (*n*)	% (*n*)	% (*n*)	% (*n*)	% (*n*)	% (*n*)	% (*n*)	% (*n*)	% (*n*)	% (*n*)
J01DD04	Watch	Ceftriaxone	6.1 (4)	30.0 (65)	24.4 (69)	-	13.6 (3)	2.4 (1)	25.8 (24)	36.5 (31)	57.1 (4)	18.8 (6)
J01MA12	Watch	Levofloxacin	-	12.4 (27)	9.5 (27)	-	-	-	20.4 (19)	4.7 (4)	-	12.5 (4)
J01FF01	Access	Clindamycin	4.6 (3)	10.6 (23)	9.2 (26)	-	13.6 (3)	-	5.4 (5)	17.7 (15)	14.3 (1)	6.3 (2)
J01DH02	Watch	Meropenem	4.6 (3)	8.8 (19)	7.8 (22)	-	-	7.1 (3)	8.6 (8)	4.7 (4)	-	21.9 (7)
J01GB06	Access	Amikacin	21.2 (14)	3.7 (8)	7.8 (22)	-	4.6 (1)	31.0 (13)	3.2 (3)	4.7 (4)	-	3.1 (1)
J01XD01	Access	Metronidazole	1.5 (1)	8.8 (19)	7.1 (20)	-	-	2.4 (1)	6.5 (6)	12.9 (11)	-	6.3 (2)
J01CA01	Access	Ampicillin	24.2 (16)	-	5.7 (16)	50.0 (1)	18.2 (4)	26.2 (11)	-	-	-	-
J01DD01	Watch	Cefotaxime	7.6 (5)	4.6 (10)	5.3 (15)	-	4.6 (1)	9.5 (4)	5.4 (5)	4.7 (4)	14.3 (1)	-
J01XA01	Watch	Vancomycin	6.1 (4)	4.6 (10)	4.9 (14)	-	-	9.5 (4)	5.4 (5)	3.5 (3)	-	6.3 (2)
J01DB03	Access	Cefalotin	13.6 (9)	0.5 (1)	3.5 (10)	50.0 (1)	36.4 (8)	-	1.1 (1)	-	-	-
J01DH51	Watch	Imipenem, cilastatin	-	4.6 (10)	3.5 (10)	-	-	-	7.5 (7)	2.4 (2)	-	3.1 (1)
J01DB01	Access	Cefalexin	3.0 (2)	1.4 (3)	1.8 (5)	-	9.1 (2)	-	-	2.4 (2)	14.3 (1)	-
J01FA09	Watch	Clarithromycin	-	1.8 (4)	1.4 (4)	-	-	-	2.2 (2)	-	-	6.3 (2)
J01MA02	Watch	Ciprofloxacin	1.5 (1)	1.4 (3)	1.4 (4)	-	-	2.4 (1)	1.1 (1)	2.4 (2)	-	-
J01EE01	Access	Sulfamethoxazole, trimethoprim	-	1.4 (3)	1.1 (3)	-	-	-	2.2 (2)	-	-	3.1 (1)
J01GB03	Access	Gentamicin	1.5 (1)	0.9 (2)	1.1 (3)	-	-	2.4 (1)	-	1.2 (1)	-	3.1 (1)
J01DE01	Watch	Cefepime	1.5 (1)	0.5 (1)	0.7 (2)	-	-	2.4 (1)	-	-	-	3.1 (1)
J01AA02	Access	Doxycycline	-	0.9 (2)	0.7 (2)	-	-	-	2.2 (2)	-	-	-
J01CR01	Access	Ampicillin, sulbactam	3.0 (2)	-	0.7 (2)	-	-	4.8 (2)	-	-	-	-
J01CA12	Watch	Piperacillin, tazobactam	-	0.5 (1)	0.4 (1)	-	-	-	-	-	-	3.1 (1)
J01XB01	Reserve	Colistin	-	0.5 (1)	0.4 (1)	-	-	-	-	-	-	3.1 (1)
J01DD02	Watch	Ceftazidime	-	0.5 (1)	0.4 (1)	-	-	-	1.1 (1)	-	-	-
J01CF01	Access	Dicloxacillin	-	0.5 (1)	0.4 (1)	-	-	-	-	1.2 (1)	-	-
J01MA14	Watch	Moxifloxacin	-	0.5 (1)	0.4 (1)	-	-	-	1.1 (1)	-	-	-
J01FA01	Watch	Erythromycin	-	0.5 (1)	0.4 (1)	-	-	-	-	1.2 (1)	-	-
J01GB05	Watch	Neomycin	-	0.5 (1)	0.4 (1)	-	-	-	1.1 (1)	-	-	-
		**Total APs**	100 (66)	100 (217)	100 (283)	3.1 (2)	33.3 (22)	63.6 (42)	42.9 (93)	39.2 (85)	3.2 (7)	14.7 (32)
Access APs			72.7 (48)	28.6 (62)	38.9 (110)	100 (2)	81.8 (18)	66.7 (28)	20.4 (19)	40.0 (34)	28.6 (2)	21.9 (7)
Watch APs			27.3 (18)	71.4 (154)	60.8 (172)	-	18.2 (4)	33.3 (14)	79.6 (74)	60.0 (51)	71.4 (5)	75.0 (24)
Reserve APs			-	0.5 (1)	0.4 (1)	-	-	-	-	-	-	3.1 (1)

Notes:

*No patients were found admitted in H1 surgical wards.

**Abbreviations**: H1: Women’s specialty hospital, H2: General referral hospital. MED: Medical service wards, SUR: Surgical service wards, MIX: Mixed service wards, ICUs: Intensive care units, APs: Antibiotic prescriptions, WHO AWaRe: Access, Watch, Reserve antibiotic classification (World Health Organization).

The most prescribed antibiotics were ampicillin (24.2%, *n* = 16/66), amikacin (21.2%, *n* = 14/66), and cefalotin (13.6%, *n* = 9/66) in H1, and ceftriaxone (30.0%, *n* = 65/217), levofloxacin (12.4%, *n* = 27/217), and clindamycin (10.6%, *n* = 23/217) in H2. The highest proportions of APs were observed in ICUs (63.6%, *n* = 42/66) and in the MIX wards (33.3%, *n* = 22/66) in H1, and in the MED (42.9%, *n* = 93/217) and SUR wards (39.2%, *n* = 85/217) in H2 (Table 3). The most prescribed antibiotics in these wards/units were amikacin (H1: ICUs, 31.0%, *n* = 13/42), cefalotin (H1: MIX, 36.4%, *n* = 8/22), and ceftriaxone (H2: MED, 25.8%, *n* = 24/93; SUR: 36.5%, *n* = 31/85) (Table 3).

### Major uses of antibiotics in patients

The most common indications for antibiotic use among patients included MPs (H1: 51.7%, *n* = 15/29), CAIs (H2: 42.9%, *n* = 42/98), and PrPs in both hospitals (H1: 27.6%, *n* = 8/29; H2: 23.5%, *n* = 23/98) (S2 Table).

In H1, most APs were for prophylactic indications (MPs: 47.0%, *n* = 31/66; PrPs: 22.7%, *n* = 15/66). Penicillins with extended-spectrum (35.5%, *n* = 11/31), and aminoglycosides (32.2%, *n* = 10/31) were the most frequently prescribed antibiotic classes (ATC 4) for MPs, while first-generation cephalosporins (40.0%, *n* = 6/15), and 3GC (26.7%, *n* = 4/15) were common choices for PrPs (S3 Table). APs for therapeutic indications were lower (HAIs: 18.2%, *n* = 12/66; CAIs: 12.1%, *n* = 8/66). Penicillins with extended-spectrum (25.0%, *n* = 3/12), and aminoglycosides (25.0%, *n* = 312) were frequently used to treat HAIs, while aminoglycosides (25.0%, *n* = 2/8), and 3GC (25.0%, *n* = 2/8) were commonly prescribed for CAIs (S3 Table). The antibiotics most prescribed (ATC 5) categorized by type of indication were ampicillin (MPs: 35.5%, *n* = 11/31; HAIs: 25.0%, *n* = 3/12), amikacin (MPs: 29.0%, *n* = 9/31; HAIs: 25.0%, *n* = 3/12; CAIs: 25.0%, *n* = 2/8), cefalotin (PrPs: 26.7%, *n* = 4/15), and ceftriaxone (PrPs: 20.0%, *n* = 3/15) (S4 Table).

In H2, antibiotics were predominantly utilized for therapeutic indications (HAIs: 22.6%, *n* = 49/217; CAIs: 47.0%, *n* = 102/217). The most used antibiotic classes (ATC 4) for therapeutic indications were 3GC (CAIs: 29.5%, *n* = 30/102; HAIs: 22.5%, *n* = 11/49), fluoroquinolones (CAIs: 17.7%, *n* = 18/102; HAIs: 20.4%, *n* = 10/49), and carbapenems (HAIs: 22.5%, *n* = 11/49). The 3GC were also the main class of antibiotics used for prophylactic indications (PrPs: 56.8%, *n* = 21/37; MPs: 48.3%, *n* = 14/29) (S3 Table). The most frequently prescribed antibiotics (ATC 5) categorized by type of indication were ceftriaxone (HAIs: 22.5%, *n* = 11/49; CAIs: 21.6%, *n* = 22/102; MPs: 44.8%, *n* = 13/29; PrPs: 51.4%, *n* = 19/37), levofloxacin (HAIs: 20.4%, *n* = 10/49; CAIs: 15.7%, *n* = 16/102), and meropenem (HAIs: 16.3%, *n* = 8/49; MPs: 10.3%, *n* = 3/29) (S4 Table).

### Use of antibiotics according to the WHO AWaRe classification

**Fig 2:**
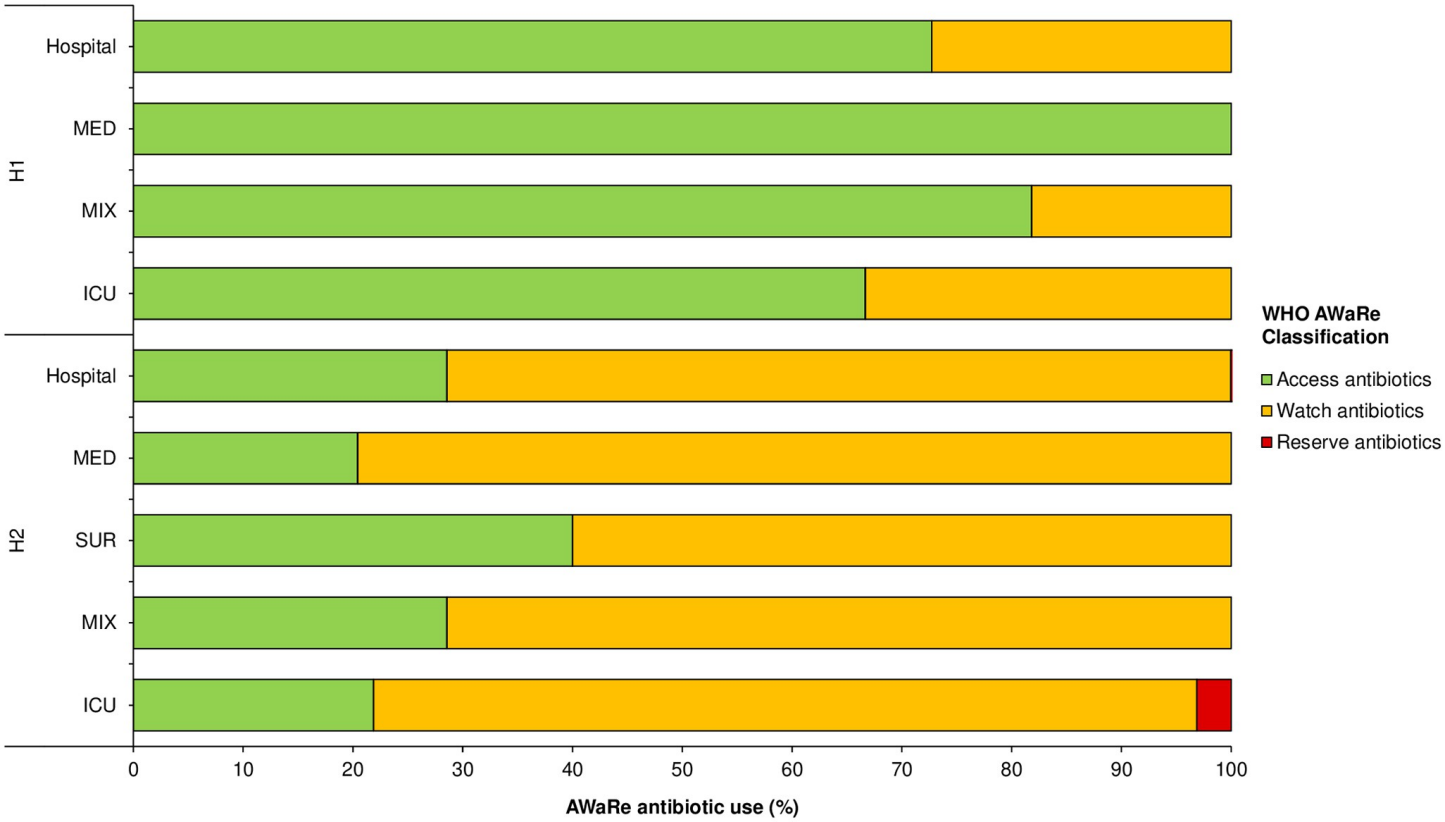
Proportion of antibiotic use according to the WHO AWaRe classification in the hospitals and their respective wards/units.

Of the 14 different antibiotics prescribed in H1, eight belonged to the Access group (Access antibiotics), while the other six belonged to the Watch group (Watch antibiotics). No APs of the Reserve group (Reserve antibiotics) were observed in H1. Conversely, among the 24 different antibiotics prescribed in H2, 14 of them were Watch antibiotics, nine were Access antibiotics, and only one was a Reserve antibiotic (Fig 2 and Table 3).

In H1, 72.7% (*n* = 48/66) of the APs were of Access antibiotics, mainly ampicillin (24.2%, *n* = 16/66), amikacin (21.2%, *n* = 14/66), and cefalotin (13.6%, *n* = 9/66). Access antibiotics were also the most prescribed agents in wards/units of H1 (MED: 100%, *n* = 2/2; MIX: 81.8%, *n* = 18/22; ICUs: 66.7%, *n* = 28/42) (Table 3). Watch antibiotics accounted for 27.3% (*n* = 18/66) of total APs in H1, mainly comprising cefotaxime (7.6%, *n* = 5/66), vancomycin (6.1%, *n* = 4/66), and ceftriaxone (6.1%, *n* = 4/66) (Table 3). Watch antibiotics accounted for 18.2% (*n* = 4/22) and 33.3% (*n* = 14/42) of APs in the MIX wards and ICUs, respectively (Fig 2 and Table 3).

Most of the APs in H2 were of Watch antibiotics (71.4%, *n* = 154/217). Ceftriaxone (30.0%, *n* = 65/217), levofloxacin (12.4%, *n* = 27/217), and meropenem (8.8%, *n* = 18/217) were the most prescribed Watch antibiotics, for both therapeutic and prophylactic indications. The Watch antibiotics were also the most frequently prescribed in all the wards/units of H2 (MED: 79.6%, *n* = 74/93; SUR: 60.0%, *n* = 51/85; MIX: 71.4%, *n* = 5/7; ICUs: 75.0%, *n* = 24/32) (Table 3). Access antibiotics accounted for 28.6% (*n* = 62/217) of the total APs in H2, mainly comprising clindamycin (10.6%, *n* = 23/217), metronidazole (8.8%, *n* = 19/217), and amikacin (3.7%, *n* = 8/217). Only one AP of a Reserve antibiotic (colistin) was identified in H2 (Fig 2 and Table 3).

### Antibiotics for preoperative prophylaxis

Multiple doses of antibiotics for more than 24 hours were administered to all the patients with PrPs in H2, and most of them in H1 (93.3%, *n* = 14/15). In H1, ceftriaxone (21.4%, *n* = 3/14) and cefalotin (21.4%, *n* = 3/14) were the predominant antibiotics used in these PrPs regimens, whereas in H2, half of the cases involved ceftriaxone (51.4%, *n* = 19/37) (Table 4).

**Table 4:** Antibiotic prescriptions for preoperative prophylaxis of multiple doses for more than one day.

		Hospital	
		H1*	H2
ATC 5 code	Antibiotics	% (*n*)	% (*n*)
J01DD04	Ceftriaxone	21.4 (3)	51.4 (19)
J01FF01	Clindamycin	14.3 (2)	10.8 (4)
J01XD01	Metronidazole	7.1 (1)	10.8 (4)
J01DB03	Cefalotin	21.4 (3)	-
J01DB01	Cefalexin	14.3 (2)	5.4 (2)
J01GB06	Amikacin	-	10.8 (4)
J01DD01	Cefotaxime	7.1 (1)	5.4 (2)
J01XA01	Vancomycin	7.1 (1)	-
J01DH02	Meropenem	-	2.7 (1)
J01MA02	Ciprofloxacin	-	2.7 (1)
J01CA01	Ampicillin	7.1 (1)	-
**Total prescriptions (*n*)**		14	37

**Notes:** No single-dose antibiotic prescriptions for preoperative prophylaxis were identified in hospitals.

*A single prescription of multiple doses of cefalotin per one day was identified.

**Abbreviations**: H1: Women’s specialty hospital, H2: General referral hospital.

### Microbiology and resistance profiles

Microbial culture results were available in 44.8% (H1, *n* = 13/29), and 21.4% (H2, *n* = 21/98) of the patients’ clinical records. Bacterial growth was observed in 38.5% (H1, *n* = 5/13) and 52.4% (H2, *n* = 11/21) of the cases. The main origin of microbial cultures was blood (H1: 92.3%, *n* = 12/13; H2: 28.6%, *n* = 6/21), surgical sites (H2: 23.8%, *n* = 5/21), sputum (H2: 14.3%, *n* = 3/21), and urine (H1: 7.7%, *n* = 1/13, H2: 14.3%, *n* = 3/21) (S5 Table). MDR isolates of *Klebsiella pneumoniae* and *Pseudomonas aeruginosa* were detected in both hospitals. In H2, MDR isolates of *Acinetobacter baumannii*, *Enterobacter cloacae*, and *Escherichia coli* were also identified (S6 Table).

## Discussion

This study highlights the extensive use of broad-spectrum antibiotics in two public secondary care hospitals in Mexico, primarily for empirical and parenteral therapies, which frequently lack post-prescription reviews. The prevalence of antibiotic use observed in this study exceeds the rates reported in other PPS studies conducted in various healthcare settings across Latin America (36.8%) [17], the USA (49.9%) [35], and Mexico (51.5% to 61.5%) [20,21,36]. However, it is still lower than the estimates documented in secondary care hospitals located in small cities (78.9%) [37]. Our findings align with antibiotic prescribing patterns observed in secondary care hospitals, particularly in the use of penicillins with extended-spectrum and first-generation cephalosporins for obstetrics and gynecology, as well as 3GC and fluoroquinolones for treating community infections or PrPs [38]. The differences in predominant antibiotic classes used across hospitals can be attributed to a variety of clinical diagnoses, the range of medical and surgical procedures performed, and patient’s condition severity. In specialized women’s healthcare institutions, antibiotics are frequently prescribed for deliveries, cesarean sections or special neonatal care, as evidenced by H1. The judicious selection of safe antibiotics for women during and after pregnancy, as well as for their newborns, may contribute to explaining the proportion of Access antibiotics prescribed in this hospital, surpassing the WHO target on these accounting for 60% of antibiotic consumption [34]. Additionally, the prevalence of neonates with suspected sepsis, treated with ampicillin and amikacin, alongside the frequent use of first-choice antibiotics, such as cefalotin, for obstetric prophylaxis, may further contribute to the utilization of Access antibiotics in H1 [39]. Conversely, more than 70% of APs in H2 were Watch antibiotics; a proportion nearly identical to that observed in other Mexican hospitals [40]. The elevated use of Watch antibiotics can be attributed to more severe medical conditions in general healthcare facilities, as evidenced by the frequency of patients with intra-abdominal sepsis, urinary tract infections, and pneumonias in H2. Regardless, Watch antibiotics should always be used cautiously and targeted to specific clinical infections, as they carry a higher risk of ARM selection, and patient toxicity compared to Access antibiotics [41]. A comprehensive systematic review and meta-analysis reveal that exposure to Watch antibiotics, such as 3GC and fluoroquinolones, increases significantly the risk of colonization or infection by extended-spectrum β-lactamase (ESBL)-producing enterobacteria and methicillin-resistant *Staphylococcus aureus*, respectively [42].

The high prevalence of patients receiving PrPs or at risk of severe infections may also contribute for the significant proportion of parenteral APs observed in both hospitals, exceeding the 71.4% reported by a global study of 129 secondary care hospitals [17]. Most PrPs antibiotics are given intravenously [43]. Parenteral antibiotic therapy is also recommended for rapidly progressing infections, such as severe sepsis, because it achieves optimal therapeutic plasma levels faster than oral dosing [44]. However, intravenous antibiotic administration may also result in patient complications associated with vascular access devices, including phlebitis, extravasation injury, thrombosis, and local or systemic infections, such as bacteremia [45,46]. It is difficult to determine whether the parenteral APs identified in our PPSs were justified by specific infections or patient conditions (such as the inability to swallow, tolerate, or absorb oral formulations), or if this is primarily due to the use of ceftriaxone, amikacin, cefalotin, and other antibiotics lacking equivalent oral formulations.

The prevalence of empiric APs observed in both hospitals may also be attributed, in part, to the presence of adult and neonatal patients with sepsis and pneumonia. Prompt initiation of antibiotic treatment is crucial in these cases, even when definitive microbiological results are not yet available at the time of diagnosis [47–49]. An analysis of a large population of patients with sepsis shows that even a single one-hour delay in initiating of antibiotic therapy can significantly increase the risk of in-hospital mortality [50]. However, medical interventions based solely on clinical experience can also carry risks, as evidenced by a large systematic review that found that up to 80% of empiric antibiotic therapies for serious infections, including bacteremia, pneumonia, sepsis, or septic shock may differ substantially from clinical treatment guidelines [51]. This is compounded by a low rate of microbial cultures and antibiograms in both hospitals that hinders etiologic confirmation of infections, the evaluation of the appropriateness of initial empirical therapies, and the recognition of local patterns of ARM. It should also be noted that most of the APs examined remained unchanged from their initiation dates. This may be due to deficiencies in the implementation of post-prescription reviews or their documentation in the clinical records. Consequently, prescribers are overlooking opportunities to assess whether antibiotic therapies should be adjusted according to microbiological findings, switched to the oral route, or discontinued [52,53]. A growing body of evidence demonstrates that short courses of antibiotics are effective in achieving clinical and microbiological resolution of common infections, such as pneumonia, urinary tract infections, and intra-abdominal infections, without increasing the risk of relapse or mortality [54–57]. A timely switch from parenteral to oral antibiotic therapy can also benefit patients by reducing the length of hospital stay and the incidence of adverse drug reactions, while still maintaining comparable therapeutic outcomes [58–60].

The lack of post-prescription reviews for antibiotics may also lead to the use of multiple doses for more than 24 hours for PrPs, instead of following the general protocol of a single dose 60 minutes before surgical incision. An appropriate PrPs protocol in obstetrics and gynecology is essential to reduce postpartum infectious morbidity, mitigate changes in the microbial composition of breast milk [61], and reduce the prevalence of ESBL-coding genes in the neonatal gastrointestinal tract [62,63]. However, we cannot exclude the possibility that some of the prolonged PrPs regimens corresponded to patients who were considered at high risk for postoperative infection [64]. Some of these patients may have received PrPs with ceftriaxone instead of first-line antibiotics such as first-generation cephalosporins.

The absence of formally established ASPs or strategies to optimize antibiotic use in the surveyed hospitals may be partly responsible for our findings. The lack of capacity to optimize antibiotic prescribing can be a common scenario in smaller hospitals [65,66], mainly due to financial, human, and IT resource constraints, compounded by the workload of healthcare personnel [26,67]. At the same time, global consumption of priority antibiotics in the healthcare sector is increasing significantly each year, mainly in hospitals in LMICs and upper-middle-income countries [40,68]. Our study highlights the urgent need to implement antibiotic surveillance measures in public secondary care hospitals as a key element to improve clinical practice standards and mitigating the ongoing threat of ARM in Mexican healthcare settings. PPSs are valuable tools for achieving this goal.

The strength of our study lies in the use of a standardized methodology and a robust data collection process, achieved through data collection forms with quality controls, a trained team of observers coordinated by the principal investigators, and the implementation of pilot studies in both hospitals.

## Limitations

The findings of this study do not necessarily reflect antibiotic use in other public secondary care hospitals in Mexico.

Our study is subject to limitations inherent in the cross-sectional design of the PPS methodology, which restricts the ability to determine daily variability or trends in antibiotic use, the final duration of antibiotic therapies, and the rate of de-escalation rate. The appropriateness of therapies could also not be evaluated, as medical records did not specify which antibiotic was prescribed for each clinical diagnosis. Furthermore, the PPS methodology does not allow the identification of institutional factors that may influence antibiotic prescribing patterns.

## Conclusions

This study shows high prevalence rates of antibiotic use and variations in the most commonly used classes of antibiotics in public secondary care hospitals in Mexico, while also identifying shared practices in the prescription of broad-spectrum antibiotics. Our findings underscore the urgent need to implement antibiotic surveillance measures in Mexican public hospitals as a key element to improve clinical practice standards and mitigate the threat of AMR. Surveillance based on PPSs enables the identification of specific targets for optimizing antibiotic use according to the healthcare needs of patients in each hospital, as well as facilitating their participation in comparative evaluations across hospitals.

## Supporting information

S1 Table Clinical diagnoses and prophylactic indications for patients with active antibiotic prescriptions. (DOCX)

S2 Table Characteristics of antibiotic prescriptions in the hospitals and their respective wards/units. (DOCX)

S3 Table Classes of antibiotics prescribed in the hospitals by type of indication. (DOCX)

S4 Table Antibiotics prescribed in the hospitals by type of indication. (DOCX)

S5 Table Characteristics of microbial cultures and AST results. (DOCX)

S6 Table AST results of isolated resistant bacteria. (DOCX)

## References

[1] Gaynes R. The discovery of penicillin—new insights after more than 75 years of clinical use. Emerg Infect Dis. 2017;23: 849–853. doi: 10.3201/eid2305.161556

[2] Holmes AH, Moore LSP, Sundsfjord A, Steinbakk M, Regmi S, Karkey A, et al. Understanding the mechanisms and drivers of antimicrobial resistance. The Lancet. 2016;387: 176–187. doi: 10.1016/S0140-6736(15)00473-0 26603922

[3] Murray CJ, Ikuta KS, Sharara F, Swetschinski L, Robles Aguilar G, Gray A, et al. Global burden of bacterial antimicrobial resistance in 2019: a systematic analysis. The Lancet. 2022;399: 629–655. doi: 10.1016/S0140-6736(21)02724-0 35065702 PMC8841637

[4] O’Neill J. Review on Antimicrobial Resistance, Tackling drug-resistant infections globally: final report and recommendations. Government of the United Kingdom. 2016.

[5] Magill SS, O’Leary E, Ray SM, Kainer MA, Evans C, Bamberg WM, et al. Assessment of the Appropriateness of Antimicrobial Use in US Hospitals. JAMA Netw Open. 2021;4. doi: 10.1001/jamanetworkopen.2021.2007 33734417 PMC7974639

[6] Tamma PD, Avdic E, Li DX, Dzintars K, Cosgrove SE. Association of adverse events with antibiotic use in hospitalized patients. JAMA Intern Med. 2017;177: 1308–1315. doi: 10.1001/jamainternmed.2017.1938 28604925 PMC5710569

[7] Marchaim D, Chopra T, Bhargava A, Bogan C, Dhar S, Hayakawa K, et al. Recent Exposure to Antimicrobials and Carbapenem-Resistant Enterobacteriaceae: The Role of Antimicrobial Stewardship. Infect Control Hosp Epidemiol. 2012;33: 817–830. doi: 10.1086/666642 22759550 PMC4370272

[8] de Kraker MEA, Davey PG, Grundmann H. Mortality and hospital stay associated with resistant Staphylococcus aureus and Escherichia coli bacteremia: Estimating the burden of antibiotic resistance in Europe. PLoS Med. 2011;8: e1001104. doi: 10.1371/journal.pmed.1001104 22022233 PMC3191157

[9] Davey P, Marwick CA, Scott CL, Charani E, McNeil K, Brown E, et al. Interventions to improve antibiotic prescribing practices for hospital inpatients. Cochrane Database of Systematic Reviews. 2017; Issue 2. Art. No.: CD003543. doi: 10.1002/14651858.CD003543.pub4 28178770 PMC6464541

[10] General Health Council. Agreement declaring the mandatory nature of the National Strategy for action against Antimicrobial Resistance. In: https://dof.gob.mx/nota_detalle.php?codigo=5525043&fecha=05/06/2018#gsc.tab=0. 5 Jun 2018.

[11] Amabile-Cuevas CF. Antibiotic usage and resistance in Mexico: an update after a decade of change. J Infect Dev Ctries. 2021;15: 442–449. doi: 10.3855/jidc.13467 33956642

[12] World Health Organization. Antimicrobial stewardship programmes in health-care facilities in low- and middle-income countries: a practical toolkit. In: Geneva: World Health Organization [Internet]. Geneva; 2019 [cited 4 Sep 2024]. https://iris.who.int/bitstream/handle/10665/329404/9789241515481-eng.pdf?sequence=1.

[13] Cox JA, Vlieghe E, Mendelson M, Wertheim H, Ndegwa L, Villegas M V., et al. Antibiotic stewardship in low- and middle-income countries: the same but different? Clinical Microbiology and Infection. 2017;23: 812–818. doi: 10.1016/j.cmi.2017.07.010 28712667

[14] Pierce J, Apisarnthanarak A, Schellack N, Cornistein W, Maani A Al, Adnan S, et al. Global Antimicrobial Stewardship with a Focus on Low- and Middle-Income Countries. International Journal of Infectious Diseases. 2020;96: 621–629. doi: 10.1016/j.ijid.2020.05.126 32505875 PMC7271868

[15] Ansari F, Erntell M, Goossens H, Davey P. The european surveillance of antimicrobial consumption (ESAC) point-prevalence survey of antibacterial use in 20 European hospitals in 2006. Clinical Infectious Diseases. 2009;49: 1496–1504. doi: 10.1086/644617 19842976

[16] World Health Organization. WHO Methodology for Point Prevalence Survey on Antibiotic Use in Hospitals version 1.1. Geneva, Switzerland; 2018 [cited 4 Sep 2024] pp. 1–102. https://apps.who.int/iris/bitstream/handle/10665/280063/WHO-EMP-IAU-2018.01-eng.pdf?ua=1.

[17] Versporten A, Zarb P, Caniaux I, Gros MF, Drapier N, Miller M, et al. Antimicrobial consumption and resistance in adult hospital inpatients in 53 countries: results of an internet-based global point prevalence survey. Lancet Glob Health. 2018;6: e619–e629. doi: 10.1016/S2214-109X(18)30186-4 29681513

[18] Plachouras D, Kärki T, Hansen S, Hopkins S, Lyytikäinen O, Moro ML, et al. Antimicrobial use in european acute care hospitals: Results from the second point prevalence survey (PPS) of healthcare-associated infections and antimicrobial use, 2016 to 2017. Eurosurveillance. 2018;23. doi: 10.2807/1560-7917.ES.23.46.1800393 30458917 PMC6247463

[19] Hsieh J, Sati H, Ramon-Pardo P, Bruinsma N, Galas M F., Marie Rwangabwoba J, et al. 2034. Standardized Point Prevalence Survey on Antibiotic Use to Inform Antimicrobial Stewardship Strategies in the Caribbean. Open Forum Infect Dis. 2019;6: S683–4. doi: 10.1093/ofid/ofz360.1714

[20] Levy Hara G, Rojas-Cortes R, Molina León HF, Dreser Mansilla A, Alfonso Orta I, Rizo-Amezquita JN, et al. Point prevalence survey of antibiotic use in hospitals in Latin American countries. Journal of Antimicrobial Chemotherapy. 2022;77: 807–815. doi: 10.1093/jac/dkab459 34957520 PMC9092443

[21] Huerta-Gutiérrez R, Braga L, Camacho-Ortiz A, Díaz-Ponce H, García-Mollinedo L, Guzmán-Blanco M, et al. One-day point prevalence of healthcare-associated infections and antimicrobial use in four countries in Latin America. International Journal of Infectious Diseases. 2019;86: 157–166. doi: 10.1016/j.ijid.2019.06.016 31229613

[22] Zumaya-Estrada FA, Ponce-De-león-garduño A, Ortiz-Brizuela E, Tinoco-Favila JC, Cornejo-Juárez P, Vilar-Compte D, et al. Point prevalence survey of antimicrobial use in four tertiary care hospitals in Mexico. Infect Drug Resist. 2021;14: 4553–4566. doi: 10.2147/IDR.S327721 34754203 PMC8572044

[23] General Directorate of Health Information (DGIS), Ministry of Health of Mexico. Unique Health Establishment Code Catalog (CLUES). In: http://gobi.salud.gob.mx/gobi/catalogos/catalogosmaestros/ESTABLECIMIENTO_SALUD_202409.xlsx?V=2024.10.30. Sep 2024.

[24] Betran AP, Ye J, Moller AB, Souza JP, Zhang J. Trends and projections of caesarean section rates: Global and regional estimates. BMJ Glob Health. 2021;6: e005671. doi: 10.1136/bmjgh-2021-005671 34130991 PMC8208001

[25] Maternal TG, Sepsis N, Working I. The Global Maternal and Neonatal Sepsis Initiative: a call for collaboration and action by 2030. Lancet Glob Health. 2017;5: e390–e391. doi: 10.1016/S2214-109X(17)30020-7 28215849

[26] Stenehjem E, Hersh AL, Sheng X, Jones P, Buckel WR, Lloyd JF, et al. Antibiotic Use in Small Community Hospitals. Clinical Infectious Diseases. 2016;63: 1273–1280. doi: 10.1093/cid/ciw588 27694483

[27] WHO Collaborating Centre for Drug Statistics Methodology. WHO Anatomical Therapeutic Chemical (ATC) Classification. In: https://atcddd.fhi.no/atc_ddd_index/. 10 May 2024.

[28] Morelos Health Services. Women’s Hospital. Technical Data Sheets 2023. In: https://www.ssm.gob.mx/portal/diagnostico-estatal-en-salud/2023/2023-1/Hospital%20de%20la%20Mujer.pdf. 2023.

[29] Morelos Health Services. Cuernavaca General Hospital. Technical Data Sheets 2023. In: https://www.ssm.gob.mx/portal/diagnostico-estatal-en-salud/2023/2023-1/Hospital-General-de-Cuernavaca.pdf. 2023.

[30] Harris PA, Taylor R, Thielke R, Payne J, Gonzalez N, Conde JG. Research electronic data capture (REDCap)-A metadata-driven methodology and workflow process for providing translational research informatics support. J Biomed Inform. 2009;42: 377–381. doi: 10.1016/j.jbi.2008.08.010 18929686 PMC2700030

[31] Pollack LA, Plachouras D, Sinkowitz-Cochran R, Gruhler H, Monnet DL, Weber JT, et al. A Concise Set of Structure and Process Indicators to Assess and Compare Antimicrobial Stewardship Programs Among EU and US Hospitals: Results From a Multinational Expert Panel. Infect Control Hosp Epidemiol. 2016/07/15. 2016;37: 1201–1211. doi: 10.1017/ice.2016.115 27418168 PMC6533629

[32] Zumaya-Estrada FA, Alpuche-Aranda CM, Saturno-Hernandez PJ. The WHO methodology for point prevalence surveys on antibiotics use in hospitals should be improved: Lessons from pilot studies in four Mexican hospitals. International Journal of Infectious Diseases. Elsevier B.V.; 2021. pp. 13–17. doi: 10.1016/j.ijid.2021.04.079 33932602

[33] StataCorp. Stata: Release 17. Statistical Software. College Station, TX: StataCorp LLC; 2021.

[34] World Health Organization. 2019 WHO AWaRe Classification Database of Antibiotics for evaluation and monitoring of use. 2019 [cited 4 Sep 2024]. https://www.who.int/publications/i/item/WHOEMPIAU2019.11.

[35] Magill SS, Edwards JR, Beldavs ZG, Dumyati G, Janelle SJ, Kainer MA, et al. Prevalence of antimicrobial use in us acute care hospitals, may-september 2011. JAMA—Journal of the American Medical Association. 2014;312: 1438–1446. doi: 10.1001/jama.2014.12923 25291579 PMC10847977

[36] Soria-Orozco M, Padrón-Salas A, González-Mercado J de J, Villava-von der Heyde N, Valerdi-Contreras L, López-Iñiguez Á, et al. Prevalencia de uso de antimicrobianos entre pacientes hospitalizados en áreas no críticas en un hospital universitario de México. Salud Publica Mex. 2017;59: 504–505. doi: 10.21149/8465 29267640

[37] Kumar S, Shukla P, Goel P, Mishra V, Gupta A, Karuna T, et al. Point Prevalence Study (PPS) of Antibiotic Usage and Bacterial Culture Rate (BCR) among Secondary Care Hospitals of Small Cities in Central India: Consolidating Indian Evidence. J Lab Physicians. 2023;15: 259–263. doi: 10.1055/s-0042-1757585 37323604 PMC10264115

[38] Yulia R, Giovanny BE, Khansa AA, Utami SP, Alkindi FF, Herawati F, et al. The Third-Generation Cephalosporin Use in a Regional General Hospital in Indonesia. International Research Journal Of Pharmacy. 2018;9: 41–45. doi: 10.7897/2230-8407.099185

[39] World Health Organization. The WHO AWaRe (Access, Watch, Reserve) antibiotic book. 2022 [cited 4 Sep 2024]. https://www.who.int/publications/i/item/WHOEMPIAU2019.11.

[40] Pauwels I, Versporten A, Drapier N, Vlieghe E, Goossens H. Hospital antibiotic prescribing patterns in adult patients according to the WHO Access, Watch and Reserve classification (AWaRe): Results from a worldwide point prevalence survey in 69 countries. Journal of Antimicrobial Chemotherapy. 2021;76: 1614–1624. doi: 10.1093/jac/dkab050 33822971 PMC8120336

[41] Sharland M, Pulcini C, Harbarth S, Zeng M, Gandra S, Mathur S, et al. Classifying antibiotics in the WHO Essential Medicines List for optimal use—be AWaRe. The Lancet Infectious Diseases. 2018. pp. 18–20. doi: 10.1016/S1473-3099(17)30724-7 29303731

[42] Sulis G, Sayood S, Katukoori S, Bollam N, George I, Yaeger LH, et al. Exposure to World Health Organization’s AWaRe antibiotics and isolation of multidrug resistant bacteria: a systematic review and meta-analysis. Clinical Microbiology and Infection. 2022. pp. 1193–1202. doi: 10.1016/j.cmi.2022.03.014 35339675

[43] Crader MF, Varacallo M. Preoperative Antibiotic Prophylaxis. In: StatPearls [Internet]. Treasure Island (FL): StatPearls Publishing. In: https://www.ncbi.nlm.nih.gov/books/NBK442032/. Jan 2024. 28723061

[44] Levy MM, Artigas A, Phillips GS, Rhodes A, Beale R, Osborn T, et al. Outcomes of the Surviving Sepsis Campaign in intensive care units in the USA and Europe: A prospective cohort study. Lancet Infect Dis. 2012;12: 919–924. doi: 10.1016/S1473-3099(12)70239-6 23103175

[45] Liu C, Chen L, Kong D, Lyu F, Luan L, Yang L. Incidence, risk factors and medical cost of peripheral intravenous catheter-related complications in hospitalised adult patients. Journal of Vascular Access. 2022;23: 57–66. doi: 10.1177/1129729820978124 33302797

[46] Maki DG, Kluger DM, Crnich CJ. The risk of bloodstream infection in adults with different intravascular devices: A systematic review of 200 published prospective studies. Mayo Clin Proc. 2006;81: 1159–1171. doi: 10.4065/81.9.1159 16970212

[47] Fraser A, Paul M, Almanasreh N, Tacconelli E, Frank U, Cauda R, et al. Benefit of Appropriate Empirical Antibiotic Treatment: Thirty-day Mortality and Duration of Hospital Stay. American Journal of Medicine. 2006;119: 970–976. doi: 10.1016/j.amjmed.2006.03.034 17071166

[48] Prina E, Ranzani O, Torres A. Community-acquired pneumonia. Lancet. 2015;386: 1097–108. doi: 10.1016/S0140-6736(15)60733-4 26277247 PMC7173092

[49] Weinberger J, Rhee C, Klompas M. A critical analysis of the literature on time-to-antibiotics in suspected sepsis. Journal of Infectious Diseases. 2020;222: S110–S118. doi: 10.1093/infdis/jiaa146 32691835

[50] Ferrer R, Martin-Loeches I, Phillips G, Osborn TM, Townsend S, Dellinger RP, et al. Empiric antibiotic treatment reduces mortality in severe sepsis and septic shock from the first hour: Results from a guideline-based performance improvement program. Crit Care Med. 2014;42: 1749–1755. doi: 10.1097/CCM.0000000000000330 24717459

[51] Marquet K, Liesenborgs A, Bergs J, Vleugels A, Claes N. Incidence and outcome of inappropriate in-hospital empiric antibiotics for severe infection: A systematic review and meta-analysis. Crit Care. 2015;19: 63. doi: 10.1186/s13054-015-0795-y 25888181 PMC4358713

[52] Ashiru-oredope D, Sharland M, Charani E, McNulty C, Cooke J. Improving the quality of antibiotic prescribing in the nhs by developing a new antimicrobial stewardship programme: Start smart-then focus. Journal of Antimicrobial Chemotherapy. 2012;67: i51–63. doi: 10.1093/jac/dks202 22855879

[53] McMullan BJ, Andresen D, Blyth CC, Avent ML, Bowen AC, Britton PN, et al. Antibiotic duration and timing of the switch from intravenous to oral route for bacterial infections in children: systematic review and guidelines. The Lancet Infectious Diseases. 2016. pp. e139–e152. doi: 10.1016/S1473-3099(16)30024-X 27321363

[54] Spellberg B. The new antibiotic mantra-"shorter is better". JAMA Internal Medicine. 2016. pp. 1254–1255. doi: 10.1001/jamainternmed.2016.3646 27455385 PMC5233409

[55] Uranga A, Espana PP, Bilbao A, Quintana JM, Arriaga I, Intxausti M, et al. Duration of antibiotic treatment in community-acquired pneumonia: A multicenter randomized clinical trial. JAMA Intern Med. 2016;176: 1257–1265. doi: 10.1001/jamainternmed.2016.3633 27455166

[56] Yahav D, Franceschini E, Koppel F, Turjeman A, Babich T, Bitterman R, et al. Seven Versus 14 Days of Antibiotic Therapy for Uncomplicated Gram-negative Bacteremia: A Noninferiority Randomized Controlled Trial. Clinical Infectious Diseases. 2019;69: 1091–1098. doi: 10.1093/cid/ciy1054 30535100

[57] Royer S, Demerle KM, Dickson RP, Prescott HC. Shorter versus longer courses of antibiotics for infection in hospitalized patients: A systematic review and meta-analysis. Journal of Hospital Medicine. 2018. pp. 336–342. doi: 10.12788/jhm.2905 29370318 PMC5945333

[58] Carratalà J, Garcia-Vidal C, Ortega L, Fernández-Sabé N, Clemente M, Albero G, et al. Effect of a 3-step critical pathway to reduce duration of intravenous antibiotic therapy and length of stay in community-acquired pneumonia: A randomized controlled trial. Arch Intern Med. 2012;172: 922–928. doi: 10.1001/archinternmed.2012.1690 22732747

[59] Oosterheert JJ, Bonten MJM, Schneider MME, Buskens E, Lammers JWJ, Hustinx WMN, et al. Effectiveness of early switch from intravenous to oral antibiotics in severe community acquired pneumonia: Multicentre randomised trial. Br Med J. 2006;333: 1193–1195. doi: 10.1136/bmj.38993.560984.BE 17090560 PMC1693658

[60] Deshpande A, Klompas M, Guo N, Imrey PB, Pallotta AM, Higgins T, et al. Intravenous to Oral Antibiotic Switch Therapy among Patients Hospitalized with Community-Acquired Pneumonia. Clinical Infectious Diseases. 2023;77: 174–185. doi: 10.1093/cid/ciad196 37011018 PMC10527888

[61] Mackeen AD, Packard RE, Ota E, Berghella V, Baxter JK. Timing of intravenous prophylactic antibiotics for preventing postpartum infectious morbidity in women undergoing cesarean delivery. Cochrane Database of Systematic Reviews. 2014. p. CD009516. doi: 10.1002/14651858.CD009516.pub2 25479008 PMC11227345

[62] Hermansson H, Kumar H, Collado MC, Salminen S, Isolauri E, Rautava S. Breast milk microbiota is shaped by mode of delivery and intrapartum antibiotic exposure. Front Nutr. 2019;6: 4. doi: 10.3389/fnut.2019.00004 30778389 PMC6369203

[63] Nogacka A, Salazar N, Suárez M, Milani C, Arboleya S, Solís G, et al. Impact of intrapartum antimicrobial prophylaxis upon the intestinal microbiota and the prevalence of antibiotic resistance genes in vaginally delivered full-term neonates. Microbiome. 2017;5: 93. doi: 10.1186/s40168-017-0313-3 28789705 PMC5549288

[64] National Institute for Health and Care Excellence (NICE). Surgical site infections: prevention and treatment. In: www.nice.org.uk/guidance/ng125. 19 Aug 2020. 31211539

[65] Pollack LA, Van Santen KL, Weiner LM, Dudeck MA, Edwards JR, Srinivasan A. Antibiotic stewardship programs in U.S. acute care hospitals: Findings from the 2014 national healthcare safety network annual hospital survey. Clinical Infectious Diseases. 2016. pp. 443–449. doi: 10.1093/cid/ciw323 27199462 PMC6537894

[66] van Dijck C, Vlieghe E, Cox JA. Antibiotic stewardship interventions in hospitals in low-and middle-income countries: A systematic review. Bulletin of the World Health Organization. 2018. pp. 266–280. doi: 10.2471/BLT.17.203448 29695883 PMC5872012

[67] Fabre V, Secaira C, Cosgrove SE, Lessa FC, Patel TS, Alvarez AA, et al. Deep Dive Into Gaps and Barriers to Implementation of Antimicrobial Stewardship Programs in Hospitals in Latin America. Clinical Infectious Diseases. 2023;77: S53–S61. doi: 10.1093/cid/ciad184 37406044 PMC10321692

[68] Klein EY, Milkowska-Shibata M, Tseng KK, Sharland M, Gandra S, Pulcini C, et al. Assessment of WHO antibiotic consumption and access targets in 76 countries, 2000–15: an analysis of pharmaceutical sales data. Lancet Infect Dis. 2021;21: 107–115. doi: 10.1016/S1473-3099(20)30332-7 32717205

