# Supplementary material for: Point prevalence survey of antibiotic use in Mexican secondary care hospitals
Source: PLoS One. 2025 Jan 3;20(1):e0315925. doi: 10.1371/journal.pone.0315925 (PMC11698459; doi:10.1371/journal.pone.0315925)
Supplement: S1 Table — (DOCX) [file pone.0315925.s001.docx]

# Point prevalence survey of antibiotic use in Mexican secondary care hospitals

# Supporting information

# S1 Table. Clinical diagnoses and prophylactic indications for patients with active antibiotic prescriptions.

|  | **Hospital** | |
| --- | --- | --- |
| **Clinical diagnosis** | **H1** | **H2** |
|  | % (*n*) | % (*n*) |
| Clinical sepsis, without laboratory confirmation | 17.1 (6) | 5.0 (6) |
| Decubitus ulcer | - | 0.8 (1) |
| Deep soft tissue infection not involving bone, not related to surgery | - | 3.4 (4) |
| Ear, nose, throat, larynx or mouth infection | - | 0.8 (1) |
| Gastrointestinal infection | - | 1.7 (2) |
| Intra-abdominal sepsis | 2.9 (1) | 9.2 (11) |
| Laboratory-confirmed bacteremia | 5.7 (2) | - |
| Lower urinary tract infection | 2.9 (1) | 9.2 (11) |
| Lung abscess, including aspergilloma | - | 0.8 (1) |
| Non-infectious diagnosis | 5.7 (2) | 8.4 (10) |
| Pneumonia or lower respiratory infections | 2.9 (1) | 16.8 (20) |
| Septic arthritis or osteomyelitis | - | 0.8 (1) |
| Septic shock | - | 0.8 (1) |
| Sexually transmitted infection | - | 0.8 (1) |
| Skin or soft tissue infection | 2.9 (1) | 5.9 (7) |
| Surgical site infection | - | 0.8 (1) |
| Upper urinary tract infection | - | 0.8 (1) |
| Ventilator associated pneumonia | - | 3.4 (4) |
| **Prophylactic indications** |  |  |
|  |  |  |
| Central nervous system prophylaxis | - | 1.7 (2) |
| Digestive tract prophylaxis | - | 6.7 (8) |
| Obstetric or gynecological infection | - | 1.7 (2) |
| Obstetric prophylaxis | 60.0 (21) | 6.7 (8) |
| Respiratory tract prophylaxis | - | 2.5 (3) |
| Skin and soft tissue prophylaxis | - | 2.5 (3) |
| Thoracic prophylaxis | - | 0.8 (1) |
| Trauma and orthopedic prophylaxis | - | 7.6 (9) |
| **Total*** | 100 (35) | 100 (119) |

**Notes:** *Patients may present more than one clinical diagnosis and/or indication for the prescription of antibiotics.

**Abbreviations:** H1: Women's specialty hospital, H2: General referral hospital.
