# Supplementary material for: Point prevalence survey of antibiotic use in Mexican secondary care hospitals
Source: PLoS One. 2025 Jan 3;20(1):e0315925. doi: 10.1371/journal.pone.0315925 (PMC11698459; doi:10.1371/journal.pone.0315925)
Supplement: S2 Table — (DOCX) [file pone.0315925.s002.docx]

# Point prevalence survey of antibiotic use in Mexican secondary care hospitals

# Supporting information

# S2 Table. Characteristics of antibiotic prescriptions in the hospitals and their respective wards/units.

| **Characteristics of antibiotic prescribing** | | **Hospital** | | **H1 wards/units*** | | | **H2 wards/units** | | | |
| --- | --- | --- | --- | --- | --- | --- | --- | --- | --- | --- |
|  |  | **H1** | **H2** | **MED** | **MIX** | **ICUs** | **MED** | **SUR** | **MIX** | **ICUs** |
|  |  | % (*n*) | % (*n*) | % (*n*) | % (*n*) | % (*n*) | % (*n*) | % (*n*) | % (*n*) | % (*n*) |
| Number of antibiotics per patient | One | 20.7 (6) | 29.6 (29) | - | 42.9 (6) | - | 25.6 (11) | 31.7 (13) | 60.0 (3) | 22.2 (2) |
|  | Two | 58.6 (17) | 39.8 (39) | 100 (1) | 57.1 (8) | 57.1 (8) | 44.2 (19) | 41.5 (17) | 40.0 (2) | 11.1 (1) |
|  | Three or more | 20.7 (6) | 30.6 (30) | - | - | 42.9 (6) | 30.2 (13) | 26.8 (11) | - | 66.7 (6) |
| Prescriptions by type of treatment | Empiric | 97.0 (64) | 98.2 (213) | 100 (2) | 100 (22) | 95.2 (40) | 97.9 (91) | 100 (85) | 100 (7) | 93.8 (30) |
|  | Target | 3.0 (2) | 1.8 (4) | - | - | 4.8 (2) | 2.1 (2) | - | - | 6.2 (2) |
| Prescriptions by route of administration | Parenteral | 90.9 (60) | 96.8 (210) | 50.0 (1) | 77.3 (17) | 100 (42) | 96.8 (90) | 97.7 (83) | 85.7 (6) | 96.9 (31) |
|  | Oral | 9.1 (6) | 3.2 (7) | 50.0 (1) | 22.7 (5) | - | 3.2 (3) | 2.3 (2) | 14.3 (1) | 3.1 (1) |
| Patients with documented post-prescription reviews | Documented review | 17.2 (5) | 2.0 (2) | - | 7.1 (1) | 28.6 (4) | 2.3 (1) | 2.4 (1) | - | - |
|  | Non documented review | 82.8 (24) | 98.0 (96) | 100 (1) | 92.9 (13) | 71.4 (10) | 97.7 (42) | 97.6 (40) | 100 (5) | 100 (9) |
| Patients by type of indication | Hospital-associated infections | 10.3 (3) | 15.3 (15) | - | - | 21.4 (3) | 16.3 (7) | 9.8 (4) | - | 44.4 (4) |
|  | Community-acquired infections | 10.3 (3) | 42.9 (42) | - | 14.3 (2) | 7.1 (1) | 53.5 (23) | 43.9 (18) | - | 11.1 (1) |
|  | Medical prophylaxis | 51.7 (15) | 18.4 (18) | 100 (1) | 35.7 (5) | 64.3 (9) | 25.6 (11) | 7.3 (3) | 40.0 (2) | 22.2 (2) |
|  | Preoperative prophylaxis | 27.6 (8) | 23.5 (23) | - | 50.0 (7) | 7.1 (1) | 4.6 (2) | 39.0 (16) | 60.0 (3) | 22.2 (2) |
|  | – Single dose | - | - | - | - | - | - | - | - | - |
|  | – Multiple dose 1 day | 12.5 (1) | - | - | 14.3 (1) | - | - | - | - | - |
|  | – Multiple dose >1 day | 87.5 (7) | 100 (23) | - | 85.7 (6) | 100 (1) | 100 (2) | 100 (16) | 100 (3) | 100 (2) |
| Mean length of antibiotic therapies | Number of days (*n*)* | 7.4 | 7.7 | 1.0 | 1.5 | 13.8 | 7.7 | 6.8 | 3.2 | 14.0 |

**Notes:** *No patients were found admitted in H1 surgical wards.

**Abbreviations**: H1: Women's specialty hospital, H2: General referral hospital. MED: medical service wards, SUR: surgical service wards, MIX: mixed service wards, UCIs: intensive care units.
