# Supplementary material for: Point prevalence survey of antibiotic use in Mexican secondary care hospitals
Source: PLoS One. 2025 Jan 3;20(1):e0315925. doi: 10.1371/journal.pone.0315925 (PMC11698459; doi:10.1371/journal.pone.0315925)
Supplement: S3 Table — (DOCX) [file pone.0315925.s003.docx]

# Point prevalence survey of antibiotic use in Mexican secondary care hospitals

# Supporting information

# S3 Table. Classes of antibiotics prescribed in the hospitals by type of indication.

|  |  |  |  | **Indications for antibiotic prescribing** | | | | | | | |
| --- | --- | --- | --- | --- | --- | --- | --- | --- | --- | --- | --- |
|  |  | **Hospital** | | **H1** | | | | **H2** | | | |
|  |  | **H1** | **H2** | **HAIs** | **CAIs** | **MPs** | **PrPs** | **HAIs** | **CAIs** | **MPs** | **PrPs** |
| **ATC 4 code** | **Class of antibiotics** | % (*n*) | % (*n*) | % (*n*) | % (*n*) | % (*n*) | % (*n*) | % (*n*) | % (*n*) | % (*n*) | % (*n*) |
| J01DD | Third-generation cephalosporins | 13.6 (9) | 35.0 (76) | 16.7 (2) | 25.0 (2) | 3.2 (1) | 26.7 (4) | 22.5 (11) | 29.5 (30) | 48.3 (14) | 56.8 (21) |
| J01MA | Fluoroquinolones | 1.5 (1) | 14.3 (31) | 8.3 (1) | - | - | - | 20.4 (10) | 17.7 (18) | 7.0 (2) | 2.7 (1) |
| J01DH | Carbapenems | 4.5 (3) | 13.4 (29) | 8.3 (1) | 12.5 (1) | 3.2 (1) | - | 22.5 (11) | 13.8 (14) | 10.3 (3) | 2.7 (1) |
| J01GB | Other aminoglycosides | 22.7 (15) | 5.1 (11) | 25.0 (3) | 25.0 (2) | 32.2 (10) | - | 6.1 (3) | 3.0 (3) | 3.5 (1) | 10.8 (4) |
| J01FF | Lincosamides | 4.5 (3) | 10.6 (23) | - | 12.5 (1) | - | 13.3 (2) | 6.1 (3) | 13.7 (14) | 6.9 (2) | 10.8 (4) |
| J01XD | Imidazole derivatives | 1.5 (1) | 8.8 (19) | - |  | - | 6.7 (1) | 6.1 (3) | 6.9 (7) | 17.2 (5) | 10.8 (4) |
| J01CA | Penicillins with extended-spectrum | 24.2 (16) | - | 25.0 (3) | 12.5 (1) | 35.5 (11) | 6.7 (1) | - | - | - | - |
| J01DB | First-generation cephalosporins | 16.7 (11) | 1.8 (4) | - | - | 16.1 (5) | 40.0 (6) | - | 2.0 (2) | - | 5.4 (2) |
| J01XA | Glycopeptides | 6.1 (4) | 4.6 (10) | 8.3 (1) | 12.5 (1) | 3.2 (1) | 6.7 (1) | 4.1 (2) | 7.8 (8) | - | - |
| J01FA | Macrolides | - | 2.3 (5) | - | - | - | - | 2.0 (1) | 3.0 (3) | 3.5 (1) | - |
| J01CR | Combinations of penicillins, incl. beta-lactams inhibitors | 3.0 (2) | 0.5 (1) | - | - | 6.5 (2) | - | 2.0 (1) | - | - | - |
| J01EE | Combinations of sulfonamides, trimetoprim | - | 1.4 (3) | - | - | - | - | 2.0 (1) | 2.0 (2) | - | - |
| J01DE | Fourth-generation cephalosporins | 1.5 (1) | 0.5 (1) | 8.3 (1) | - | - | - | 2.0 (1) | - | - | - |
| J01AA | Tetracyclines | - | 0.9 (2) | - | - | - | - | 2.0 (1) | - | 3.5 (1) | - |
| J01CF | Beta-lactamase resistant penicillins | - | 0.5 (1) | - | - | - | - | - | 1.0 (1) | - | - |
| J01XB | Polymyxins | - | 0.5 (1) | - | - | - | - | 2.0 (1) | - | - | - |
| **Total APs** | | 100 (66) | 100 (217) | 18.2 (12) | 12.1 (8) | 47.0 (31) | 22.7 (15) | 22.6 (49) | 47.0 (102) | 13.4 (29) | 17.1 (37) |

**Abbreviations**: H1: Women's specialty hospital, H2: General referral hospital. HAIs: hospital-acquired infections, CAIs: community-acquired infections, PrP: preoperative prophylaxis, MPs: medical prophylaxis, APs: antibiotic prescriptions.
