# Supplementary material for: Point prevalence survey of antibiotic use in Mexican secondary care hospitals
Source: PLoS One. 2025 Jan 3;20(1):e0315925. doi: 10.1371/journal.pone.0315925 (PMC11698459; doi:10.1371/journal.pone.0315925)
Supplement: S4 Table — (DOCX) [file pone.0315925.s004.docx]

# Point prevalence survey of antibiotic use in Mexican secondary care hospitals

# Supporting information

# S4 Table. Antibiotics prescribed in the hospitals by type of indication.

|  |  | **Hospital** | | **Global** | **H1** | **H2** | **H1** | **H2** | **H1** | **H2** | **H1** | **H2** |
| --- | --- | --- | --- | --- | --- | --- | --- | --- | --- | --- | --- | --- |
| **Antibiotics prescribed** | | **H1** | **H2** |  | **HAIs** | | **CAIs** | | **MPs** | | **PrPs** | |
| ATC 5 code | Antibiotic | % (*n*) | % (*n*) | % (*n*) | % (*n*) | % (*n*) | % (*n*) | % (*n*) | % (*n*) | % (*n*) | % (*n*) | % (*n*) |
| J01DD04 | Ceftriaxone | 6.1 (4) | 30.0 (65) | 24.4 (69) | - | 22.5 (11) | 12.5 (1) | 21.6 (22) | - | 44.8 (13) | 20.0 (3) | 51.4 (19) |
| J01MA12 | Levofloxacin | - | 12.4 (27) | 9.5 (27) | - | 20.4 (10) | - | 15.7 (16) | - | 3.5 (1) | - | - |
| J01FF01 | Clindamycin | 4.6 (3) | 10.6 (23) | 9.2 (26) | - | 6.1 (3) | 12.5 (1) | 13.7 (14) | - | 6.9 (2) | 13.3 (2) | 10.8 (4) |
| J01DH02 | Meropenem | 4.6 (3) | 8.8 (19) | 7.8 (22) | 8.3 (1) | 16.3 (8) | 12.5 (1) | 6.9 (7) | 3.2 (1) | 10.3 (3) | - | 2.7 (1) |
| J01GB06 | Amikacin | 21.2 (14) | 3.7 (8) | 7.8 (22) | 25.0 (3) | 4.1 (2) | 25.0 (2) | 2.0 (2) | 29.0 (9) | - | - | 10.8 (4) |
| J01XD01 | Metronidazole | 1.5 (1) | 8.8 (19) | 7.1 (20) | - | 6.1 (3) | - | 6.9 (7) | - | 17.2 (5) | 6.7 (1) | 10.8 (4) |
| J01CA01 | Ampicillin | 24.2 (16) | - | 5.7 (16) | 25.0 (3) | - | 12.5 (1) | - | 35.5 (11) | - | 6.7 (1) | - |
| J01DD01 | Cefotaxime | 7.6 (5) | 4.6 (10) | 5.3 (15) | 16.7 (2) | - | 12.5 (1) | 6.9 (7) | 3.2 (1) | 3.5 (1) | 6.7 (1) | 5.4 (2) |
| J01XA01 | Vancomycin | 6.1 (4) | 4.6 (10) | 4.9 (14) | 8.3 (1) | 4.1 (2) | 12.5 (1) | 7.8 (8) | 3.2 (1) | - | 6.7 (1) | - |
| J01DB03 | Cefalotin | 13.6 (9) | 0.5 (1) | 3.5 (10) | - | - | - | 1.0 (1) | 16.1 (5) | - | 26.7 (4) | - |
| J01DH51 | Imipenem, cilastatin | - | 4.6 (10) | 3.5 (10) | - | 6.1 (3) | - | 6.9 (7) | - | - | - | - |
| J01DB01 | Cefalexin | 3.0 (2) | 1.4 (3) | 1.8 (5) | - | - | - | 1.0 (1) | - | - | 13.3 (2) | 5.4 (2) |
| J01FA09 | Clarithromycin | - | 1.8 (4) | 1.4 (4) | - | 2.0 (1) | - | 2.0 (2) | - | 3.5 (1) | - | - |
| J01MA02 | Ciprofloxacin | 1.5 (1) | 1.4 (3) | 1.4 (4) | 8.3 (1) | - | - | 2.0 (2) | - | - | - | 2.7 (1) |
| J01EE01 | Sulfamethoxazole, trimethoprim | - | 1.4 (3) | 1.1 (3) | - | 2.0 (1) | - | 2.0 (2) | - | - | - | - |
| J01GB03 | Gentamicin | 1.5 (1) | 0.9 (2) | 1.1 (3) | - | 2.0 (1) | - | 1.0 (1) | 3.2 (1) | - | - | - |
| J01DE01 | Cefepime | 1.5 (1) | 0.5 (1) | 0.7 (2) | 8.3 (1) | 2.0 (1) | - | - | - | - | - | - |
| J01AA02 | Doxycycline | - | 0.9 (2) | 0.7 (2) | - | 2.0 (1) | - | - | - | 3.5 (1) | - | - |
| J01CR01 | Ampicillin, sulbactam | 3.0 (2) | - | 0.7 (2) | - | - | - | - | 6.5 (2) | - | - | - |
| J01CA12 | Piperacillin, tazobactam | - | 0.5 (1) | 0.4 (1) | - | 2.0 (1) | - | - | - | - | - | - |
| J01XB01 | Colistin | - | 0.5 (1) | 0.4 (1) | - | 2.0 (1) | - | - | - | - | - | - |
| J01DD02 | Ceftazidime | - | 0.5 (1) | 0.4 (1) | - | - | - | 1.0 (1) | - | - | - | - |
| J01CF01 | Dicloxacillin | - | 0.5 (1) | 0.4 (1) | - | - | - | 1.0 (1) | - | - | - | - |
| J01MA14 | Moxifloxacin | - | 0.5 (1) | 0.4 (1) | - | - | - | - | - | 3.5 (1) | - | - |
| J01FA01 | Erythromycin | - | 0.5 (1) | 0.4 (1) | - | - | - | 1.0 (1) | - | - | - | - |
| J01GB05 | Neomycin | - | 0.5 (1) | 0.4 (1) | - | - | - | - | - | 3.5 (1) | - | - |
|  | **Total APs** | 100 (66) | 100 (217) | 283 | 18.2 (12) | 22.6 (49) | 12.1 (8) | 47.0 (102) | 47.0 (31) | 13.4 (29) | 22.7 (15) | 17.0 (37) |

**Abbreviations**: H1: Women's specialty hospital, H2: General referral hospital. HAIs: hospital-acquired infections, CAIs: community-acquired infections, PrPs: preoperative prophylaxis, MPs: medical prophylaxis. APs: antibiotic prescriptions.
