# Supplementary material for: Point prevalence survey of antibiotic use in Mexican secondary care hospitals
Source: PLoS One. 2025 Jan 3;20(1):e0315925. doi: 10.1371/journal.pone.0315925 (PMC11698459; doi:10.1371/journal.pone.0315925)
Supplement: S5 Table — (DOCX) [file pone.0315925.s005.docx]

# Point prevalence survey of antibiotic use in Mexican secondary care hospitals

# Supporting information

# S5 Table. Characteristics of microbial cultures and AST results.

|  | **Hospital** | |
| --- | --- | --- |
|  | **H1** | **H2** |
|  | % (*n*) | % (*n*) |
| Patients with at least one microbial culture | 44.8 (13) | 21.4 (21) |
| Microbial cultures with bacterial growth | 38.5 (5) | 52.4 (11) |
| **Anatomical origin of microbial cultures** |  |  |
| – Abscess | - | 4.8 (1) |
| – Blood | 92.3 (12) | 28.6 (6) |
| – Catheter | - | 4.8 (1) |
| – Empyema | - | 4.8 (1) |
| – Sputum | - | 14.3 (3) |
| – Surgical site | - | 23.8 (5) |
| – Urine | 7.7 (1) | 14.3 (3) |
| – Not specified | - | 4.8 (1) |
| **Isolated bacteria species** |  |  |
| Gram negatives |  |  |
| *– Acinetobacter baumannii* | - | 10 (1)^M^ |
| *– Burkholderia cepacia* | - | 10 (1)^M^ |
| *– Enterobacter cloacae* | - | 10 (1)^M^ |
| *– Escherichia coli* | - | 20 (2)^M^ |
| *– Klebsiella pneumoniae* | 33.3 (2)^M^ | 10 (1)^M^ |
| *– Pseudomonas aeruginosa* | 16.7 (1)^M^ | 40 (4)^M^ |
| Gram positives |  |  |
| *– Gram-positive cocci, not specified* | 16.7 (1)^S^ | - |
| *– Staphylococcus aureus* | - | 10 (1)^S^ |
| *– Staphylococcus epidermidis* | 16.7 (1)^M^ | 10 (1)^S^ |
| *– Staphylococcus saprophiticcus* | - | 10 (1)^M^ |
| *– Streptococcus agalactia* | 16.7 (1)^R^ | 10 (1)^S^ |
| **Total bacterial isolations** | 6 (100) | 14 (100) |

**Abbreviations**: H1: Women's specialty hospital, H2: General referral hospital. AST: antimicrobial susceptibility testing, ^M^: multidrug-resistant bacteria, ^R^: resistant bacteria, ^S^: susceptible bacteria.
