# Supplementary material for: Point prevalence survey of antibiotic use in Mexican secondary care hospitals
Source: PLoS One. 2025 Jan 3;20(1):e0315925. doi: 10.1371/journal.pone.0315925 (PMC11698459; doi:10.1371/journal.pone.0315925)
Supplement: S6 Table — (DOCX) [file pone.0315925.s006.docx]

# Point prevalence survey of antibiotic use in Mexican secondary care hospitals

# Supporting information

# S6 Table. AST results of isolated resistant bacteria.

| **Hospital** | **Bacterial species** | **ATC 5 code - Antibiotics** (>1 AST result) | **ATC 3 code - Antibiotic groups** (number of AST results) |
| --- | --- | --- | --- |
|  |  |  |  |
| **H1** | *Klebsiella pneumoniae* | J01CR02 - Amoxicillin, beta-lactamase inhibitor (2) \| J01CR01 - Ampicillin, sulbactam (2) \| J01CA01 - Ampicillin (2) \| J01DB03 - Cefalotin (2) \| J01DE01 - Cefepime \| J01DD01 - Cefotaxime (2) \| J01DD02 - Ceftazidime (2) \| J01DD04 - Ceftriaxone (2) \| J01MA02 - Ciprofloxacin (2) \| J01GB03 - Gentamicin (2) \| J01XE01 - Nitrofurantoin \| J01EE01 - Sulfamethoxazole, trimethoprim (2) | J01C - Beta-lactam antibacterials, penicillins (6) \| J01D - Other Beta-lactam antibacterials (9) \| J01M - Quinolone antibacterials (2) \| J01G - Aminoglycoside antibacterials (2) \| J01X - Other antibacterials (1) \| J01E - Sulfonamides and trimethoprim (2) |
|  | *Pseudomonas aeruginosa* | J01CR02 - Amoxicillin, beta-lactamase inhibitor \| J01DB03 - Cefalotin \| J01DE01 - Cefepime \| J01DD01 - Cefotaxime \| J01DD02 - Ceftazidime \| J01DD04 - Ceftriaxone \| J01DH02 - Meropenem \| J01MA02 - Ciprofloxacin \| J01MA06 - Norfloxacin \| J01GB03 – Gentamicin | J01C - Beta-lactam antibacterials, penicillins (1) \| J01D - Other Beta-lactam antibacterials (6) \| J01M - Quinolone antibacterials (2) \| J01G - Aminoglycoside antibacterials (1) |
|  | *Staphylococcus epidermidis* | J01MA02 - Ciprofloxacin \| J01MA12 - Levofloxacin \| J01MA14 - Moxifloxacin \| J01FA01 - Erythromycin \| J01GB03 - Gentamicin \| J01CF04 – Oxacillin | J01M - Quinolone antibacterials (3) \| J01F - Macrolides, lincosamides and streptogramins (1) \| J01G - Aminoglycoside antibacterials (1) \| J01C - Beta-lactam antibacterials, penicillins (1) |
|  | *Streptococcus agalactiae* | J01AA07 – Tetracycline | J01A - Tetracyclines (1) |
| **H2** | *Acinetobacter baumannii* | J01CR02 - Amoxicillin, beta-lactamase inhibitor \| J01CR01 - Ampicillin, sulbactam \| J01DB03 - Cefalotin \| J01DD01 - Cefotaxime \| J01DD02 - Ceftazidime \| J01DD04 - Ceftriaxone \| J01MA02 - Ciprofloxacin \| J01XX01 - Fosfomycin \| J01GB03 - Gentamicin \| J01EE01 - Sulfamethoxazole, trimethoprim | J01C - Beta-lactam antibacterials, penicillins (2) \| J01D - Other Beta-lactam antibacterials (4) \| J01M - Quinolone antibacterials (1) \| J01X - Other antibacterials (1) \| J01G - Aminoglycoside antibacterials (1) \| J01E - Sulfonamides and trimethoprim (1) |
|  | *Burkholderia cepacia* | J01GB06 - Amikacin \| J01CR02 - Amoxicillin, beta-lactamase inhibitor \| J01DB03 - Cefalotin \| J01DD01 - Cefotaxime \| J01DD04 - Ceftriaxone \| J01XX01 - Fosfomycin \| J01GB03 - Gentamicin \| J01EE01 - Sulfamethoxazole, trimethoprim | J01G - Aminoglycoside antibacterials (1) \| J01C - Beta-lactam antibacterials, penicillins (1) \| J01D - Other Beta-lactam antibacterials (3) \| J01X - Other antibacterials (1) \| J01G - Aminoglycoside antibacterials (1) \| J01E - Sulfonamides and trimethoprim (1) |
|  | *Enterobacter cloacae* | J01CR02 - Amoxicillin, beta-lactamase inhibitor \| J01DB03 - Cefalotin \| J01DD01 - Cefotaxime \| J01DD02 - Ceftazidime \| J01DD04 - Ceftriaxone \| J01MA02 - Ciprofloxacin \| J01MA06 - Norfloxacin \| J01XX01 - Fosfomycin \| J01GB03 - Gentamicin \| J01EE01 - Sulfamethoxazole, trimethoprim | J01C - Beta-lactam antibacterials, penicillins (1) \| J01D - Other Beta-lactam antibacterials (4) \| J01M - Quinolone antibacterials (2) \| J01X - Other antibacterials (1) \| J01G - Aminoglycoside antibacterials (1) \| J01E - Sulfonamides and trimethoprim (1) |
|  | *Escherichia coli* | J01DD04 - Ceftriaxone \| J01MA02 - Ciprofloxacin (2) \| J01MA06 - Norfloxacin \| J01GB03 - Gentamicin \| J01EE01 - Sulfamethoxazole, trimethoprim | J01D - Other Beta-lactam antibacterials (1) \| J01M - Quinolone antibacterials (3) \| J01G - Aminoglycoside antibacterials (1) \| J01E - Sulfonamides and trimethoprim (1) |
|  | *Klebsiella pneumoniae* | J01CR02 - Amoxicillin, beta-lactamase inhibitor \| J01CA01 - Ampicillin \| J01DB03 - Cefalotin \| J01DD01 - Cefotaxime \| J01DD02 - Ceftazidime \| J01DD04 - Ceftriaxone \| J01DH02 - Meropenem \| J01MA02 - Ciprofloxacin \| J01XX01 - Fosfomycin \| J01XE01 - Nitrofurantoin | J01C - Beta-lactam antibacterials, penicillins (2) \| J01D - Other Beta-lactam antibacterials (5) \| J01M - Quinolone antibacterials (1) \| J01X - Other antibacterials (2) |
|  | *Pseudomonas aeruginosa* | J01GB06 - Amikacin (3) \| J01GB03 - Gentamicin (3) \| J01CR02 - Amoxicillin, beta-lactamase inhibitor (4) \| J01CR01 - Ampicillin, sulbactam \| J01DB03 - Cefalotin (2) \| J01DD01 - Cefotaxime (2) \| J01DD02 - Ceftazidime (2) \| J01DD04 - Ceftriaxone (3) \| J01DH02 - Meropenem (2) \| J01MA02 - Ciprofloxacin (3) \| J01MA06 - Norfloxacin (3) \| J01XX01 - Fosfomycin (2) | J01G - Aminoglycoside antibacterials (6) \| J01C - Beta-lactam antibacterials, penicillins (5) \| J01D - Other Beta-lactam antibacterials (11) \| J01M - Quinolone antibacterials (6) \| J01X - Other antibacterials (2) |
|  | *Staphylococcus saprophiticcus* | J01CA01 - Ampicillin \| J01MA02 - Ciprofloxacin \| \| J01MA12 - Levofloxacin \| J01MA14 - Moxifloxacin \| J01FA01 - Erythromycin \| J01GB03 - Gentamicin \| J01EE01 - Sulfamethoxazole, trimethoprim | J01C - Beta-lactam antibacterials, penicillins (1) \| J01M - Quinolone antibacterials (3) \| J01F - Macrolides, lincosamides and streptogramins (1) \| J01G - Aminoglycoside antibacterials (1) \| J01E - Sulfonamides and trimethoprim (1) |

**Abbreviations**: H1: Women's specialty hospital, H2: General referral hospital. AST: antimicrobial susceptibility testing.
